# Diabetes Stigma and Clinical Outcomes: An International Review

**DOI:** 10.1210/jendso/bvae136

**Published:** 2024-08-05

**Authors:** Kelsey B Eitel, Catherine Pihoker, Catherine E Barrett, Alissa J Roberts

**Affiliations:** Department of Pediatrics, University of Washington, Seattle, WA 98105, USA; Department of Pediatrics, University of Washington, Seattle, WA 98105, USA; Centers for Disease Control and Prevention, Atlanta, GA 30341, USA; Department of Pediatrics, University of Washington, Seattle, WA 98105, USA

**Keywords:** diabetes-related stigma, HbA1c, clinical outcomes, diabetic ketoacidosis, complications

## Abstract

Diabetes stigma is the social burden of living with diabetes. People with diabetes may experience or perceive an adverse social judgment, prejudice, or stereotype about living with diabetes at work, school, in healthcare settings, popular culture, or relationships. This review describes the methods that have been used to assess diabetes stigma, and explores the prevalence of diabetes stigma, associated sociodemographic and socioeconomic factors, cultural factors, and how diabetes stigma is associated with clinical outcomes, including HbA1c levels, diabetic ketoacidosis, severe hypoglycemia, and chronic complications, in addition to psychosocial complications in youth, adolescents, and adults with type 1 diabetes (T1D) and type 2 diabetes (T2D). The prevalence of diabetes stigma has been reported as high as 78% in adults with T1D, 70% in adults with T2D, 98% in youth and adolescents with T1D, and is unknown in youth and adolescents with T2D. Diabetes stigma has been associated with lower psychosocial functioning, decreased self-care behaviors, higher HbA1c levels, and higher frequency of diabetes complications in adults with T1D and T2D. In adolescents and young adults with T1D, diabetes stigma is associated with lower psychosocial functioning, higher HbA1c levels, and higher frequency of diabetic ketoacidosis and severe hypoglycemia episodes in addition to chronic complications. In youth and adolescents with T2D, one study demonstrated an association of diabetes stigma with lower psychosocial functioning, higher HbA1c levels, and presence of retinopathy. Gaps exist in our understanding of the mechanisms of diabetes stigma, particularly in youth and adolescents with T2D.

Diabetes stigma is an experience of exclusion, rejection, or blame due to an adverse social judgment, stereotype, or prejudice about a person with diabetes [1]. In contrast, discrimination is the unfair or prejudicial treatment of a person due to their diabetes [1]. An example of diabetes stigma is when a person with diabetes gives an insulin injection in a public space, notices other people staring at them, and then overhears a comment “It's a disgrace they are doing drugs in public.” An example of diabetes discrimination is not being able to enroll a child in daycare due to their diabetes.

People with diabetes experience diabetes stigma in school, workplaces, healthcare settings, popular culture, social media, and relationships [1-3]. People may be perceived as having diabetes through factors such as blood glucose monitoring, insulin injections, wearable diabetes technology, dietary choices or restrictions, hypoglycemic episodes, or associated comorbidities, including obesity, acanthosis nigricans, foot ulcers, or dialysis. There are several types of diabetes stigma, including experienced stigma, perceived stigma, anticipated stigma, internalized or self-stigma, and intersectional stigma in which diabetes stigma is compounded by stigma from other conditions (ie, obesity, smoking, or mental health diagnoses) or characteristics (race and ethnicity, gender, sexual identity) [1].

Diabetes stigma is a common lived experience for people with diabetes, and its consequences have become an increasing focus of research in the last 10 years. The earliest study focusing on the impact of diabetes stigma on self-management behaviors was published in 2003 [4], followed by key studies by Browne in 2013 [2] and Kato in 2014 [5]. Before 2020, there were 6 studies assessing impacts of diabetes stigma, in contrast to 22 studies since 2020. Qualitative and quantitative studies have explored the impact of diabetes stigma, demonstrating that diabetes stigma is associated with decreased self-care behaviors, decreased psychosocial functioning, and worse clinical outcomes [1]. Type 1 diabetes (T1D) and type 2 diabetes (T2D) have different etiologies and may vary in treatment plans; diabetes stigma has been associated with worse clinical outcomes in both groups [6, 7].

This review discusses the prevalence of and assessment tools for measuring diabetes stigma. Additionally, we examine associated sociodemographic, socioeconomic, and cultural factors, in addition to psychosocial and clinical outcomes that have been associated with diabetes stigma, including glycated hemoglobin (HbA1c) levels and time in recommended glucose range, diabetic ketoacidosis (DKA), severe hypoglycemia, and long-term complications such as cardiovascular disease, dyslipidemia, microalbuminuria, nephropathy, retinopathy, and neuropathy.

## Materials and Methods

We searched PubMed using the following terms: (((*diabetes stigma*) OR (*stigma*)) AND (*diabetes*)) AND (*a1c* OR *outcome* OR *glycemic control*) from 2000 to January 2, 2024. The initial search yielded 213 items. Of these, 186 were excluded because they did not discuss prevalence of diabetes stigma or did not discuss diabetes stigma and one of the following diabetes clinical outcomes: HbA1c, time in range, DKA, severe hypoglycemia, dyslipidemia, cardiovascular disease, microalbuminuria, nephropathy, retinopathy, or neuropathy. Additional references were identified by reviewing the included articles’ reference lists and were included in the review if they met the above criteria. These additional references were not found within the initial search because stigma, while part of the study, was not included in the title or abstract.

In this review we included studies that used validated diabetes stigma assessment tools, described below, as well as studies that used nonvalidated surveys and qualitative studies. Given that a variety of validated and nonvalidated measures have been used, and that there are no specific thresholds defining diabetes stigma, the presence of diabetes stigma and associated factors and outcomes are assessed and described in various ways throughout this review. Studies including prevalence data used a variety of methods, including interview-based qualitative studies [2, 3, 8, 9] and population-based studies with representative samples [6, 9-14].

## Prevalence of Diabetes Stigma

The prevalence of diabetes stigma in adults with T1D ranges from 52% to 78% [3, 6, 10], whereas the prevalence in adults with T2D ranges from 12% to 70% [2, 6, 8, 9, 11]. The largest study used an unvalidated survey and included 5422 adults with diabetes and parents of children with diabetes and found 74% of those with T1D (N = 1572) and 52% of those with T2D (N = 3850) reported that diabetes was associated with stigma [6]. In this study, for those with T1D under 18 years of age, parents of children with diabetes (13% of the T1D sample) completed the survey on behalf of their child; parents of children with diabetes were significantly more likely to perceive diabetes stigma than adults with diabetes (83% vs 74%, *P* = .006) [6].

There is less data on youth and adolescents with diabetes, but studies suggest a prevalence of 59% to 98.7% in those with T1D [12-14]. Adolescents may be especially vulnerable to perceiving or experiencing stigma at this developmental stage given their emphasis on personal identity and peer relationships [15]. The largest study (N = 380) in adolescents and young adults with T1D using a validated survey reported a stigma prevalence of 65.5% [12]. Another study, the SEARCH for Diabetes in Youth study, used a nonvalidated diabetes stigma survey and reported similar stigma scores for adolescents with T1D (mean 10.9, SD 5.4) and T2D (mean 9.8, SD 5.6), with total possible scores ranging from 5 to 30 [7]. There have been few studies, other than the aforementioned SEARCH study, examining prevalence of diabetes stigma in youth or adolescents with T2D despite the rapid growth of this population [16].

Some sociodemographic characteristics have been associated with increased experience or perception of diabetes stigma. In people with T1D, diabetes stigma is associated with female sex [6, 7, 10], lower age [10, 17], and shorter diabetes duration [6, 10]. In people with T2D, diabetes stigma is associated with female sex [6-8, 18], higher body mass index [6, 19], and use of insulin [6, 7, 20-22]. However, the associations of diabetes stigma with age in people with T2D are variable, with one study reporting higher stigma with age > 50 years [11], one study with age ≤ 60 years [23], and another study no significant difference in diabetes stigma scores based on age [18]. Similarly, findings on the association between diabetes stigma and diabetes duration are inconclusive, with some studies reporting that diabetes stigma decreases with the duration of T2D [18, 20], and another finding that diabetes stigma was lowest after initial diabetes diagnosis then gradually increased and was highest in adults diagnosed with T2D for 11 to 15 years [24].

Diabetes stigma has a high reported prevalence in all studied populations, although prevalence is unknown in youth and adolescents with T2D. Female sex has been consistently associated with increased experience and perception of diabetes stigma across all populations, which is similar to the increased burden of other psychosocial comorbidities in females with diabetes, such as depression [25], and likely due to societal and cultural factors.

## Diabetes Stigma Assessment Tools

Several diabetes stigma assessment tools exist (Table 1), although none have a defined cutoff score for high frequency or clinically significant levels of stigma [5, 22, 26-35]. The Type 1 Diabetes Stigma Assessment Scale (DSAS-1) and Type 2 Diabetes Stigma Assessment Scale (DSAS-2) are 19-item surveys initially developed in adults in Australia [26, 27], and now are translated and validated in several languages [28-32]. The DSAS-1 and DSAS-2 are scored on a 5-point Likert scale, and each have 3 subscales, with 2 subscales in common: 1) Treated Differently; and 2) Blame and Judgement [26, 27]. The third subscale for DSAS-1 is Identity Concerns, which includes possible threats to personal identity (ie, being mistaken for a drug user when injecting insulin) [26]. The DSAS-2 third subscale is Self-Stigma, which includes internalized judgments such as guilt and shame [27]. There has also been a validation of a shortened 8-item DSAS-1 [33]. The Self Stigma Scale (SSS-J) is a 39-item survey validated in adults with T2D in Japan that is scored on a 4-point Likert scale and has 3 domains (Cognitive, Affective, and Behavioral) [5]. The Kanden Institute Stigma Scale (KISS) is a validated 24-item survey that was developed in Japan for adults with T1D, T2D, gestational diabetes, and other types of diabetes [22]. It is scored on a 4-point Likert scale and has 6 subscales: Social-Enacted, Discordant-Enacted, Self-Enacted, Social-Perceived, Discordant-Perceived, and Self-Perceived [22]. The Diabetes Self Stigma Scale is a 16-item survey validated with adults with T2D in Korea and consists of 4 domains: Comparative Inability, Social Withdrawal, Self-Devaluation, and Apprehensive Feeling [34]. The Barriers to Diabetes Adherence stigma 6-item subscale is the only validated tool to assess diabetes stigma in adolescents with T1D, age 12-17 years [35]. There are no validated surveys for youth less than 12 years old or those less than 18 years old with T2D. Selection of an assessment tool may depend on age, type of diabetes, cultural factors, and language of validation.

**Table 1.** Validated diabetes stigma assessment tools

Name	Number of Items	Origin	Population	Subscales	Scoring
Type 1 Diabetes Stigma Assessment Scale (DSAS-1)	19	Australia	T1D ≥ 18 years	Treated Differently Blame and Judgement Identity Concerns	5-point Likert scale
Type 2 Diabetes Stigma Assessment Scale (DSAS-2)	19	Australia	T2D ≥ 18 years	Treated Differently Blame and Judgement Self-Stigma	5-point Likert scale
Self-Stigma Scale (SSS-J)	39	Japan	T2D ≥ 18 years	Cognitive Affective Behavioral	4-point Likert scale
Kanden Institute Stigma Scale (KISS)	24	Japan	T1D, T2D, gestational diabetes, other types of diabetes ≥ 18 years	Social-Enacted Discordant-Enacted Self-Enacted Social-Perceived Discordant-Perceived Self-Perceived	4-point Likert scale
Diabetes Self Stigma Scale	16	Korea	T2D ≥ 18 years	Comparative Inability Social Withdrawal Self-Devaluation Apprehensive Feeling	5-point Likert scale
Barriers to Diabetes Adherence stigma subscale	6	United States	T1D 12-17 years	N/A	5-point Likert scale

Abbreviations: T1D, type 1 diabetes; T2D type 2 diabetes.

## Stigma and Clinical Outcomes

### Adults With T1D

Studies in adults have consistently demonstrated an association between diabetes stigma scores and higher HbA1c levels collected by either self-reporting [6, 16, 36] or by laboratory measurement [10, 37, 38]. The largest study (n = 1594) was cross-sectional and used a validated survey [10]. For every 1-point increase in stigma score using validated surveys, the HbA1c β coefficient ranged from 0.01 to 0.33 in linear regression models [38] adjusted for age, sex, diabetes duration [10, 36], educational level [10], and number of diabetes complications [36]. One study has demonstrated a negative correlation between diabetes stigma and glucose time in recommended range [33].

There are limited data on the relationship between diabetes stigma, DKA episodes, severe hypoglycemia episodes, and chronic complications of diabetes in adults with T1D. Hansen et al showed that higher diabetes stigma scores were associated with having at least one diabetes complication [10].

### Youth and Adolescents With T1D

As in adults, diabetes stigma in youth and adolescents with T1D has been associated with a higher HbA1c level [7, 12, 13, 39, 40]. In one study, unvalidated surveys were completed by parents of children with T1D and found that the youth's mean HbA1c levels were higher when parents reported parental stigma and depression compared to parents who endorsed parental stigma but no depression [39]. Increased frequency of hypoglycemia has also been associated with diabetes stigma in adolescents with T1D [1, 7, 40]. In the largest study (N = 1255) of adolescents and young adults (age range, 10-24.9 years), which used an unvalidated survey and adjusted for a number of confounding factors (race and ethnicity, sex, treatment plan, HbA1c, age, duration of diabetes, education level, health insurance), there was an association between diabetes stigma scores and DKA episodes (β = 1.61, *P* = .0003), severe hypoglycemia episodes (β = 1.60, *P* = .0022), retinopathy (β = 1.94, *P* = .0002), and nephropathy (β = 1.16, *P* = .04) [7]. There are no published studies in youth examining glucose time in range. There are no studies to our knowledge looking at parent-reported stigma and associations with acute or chronic complications in youth with T1D.

### Adults With T2D

Several quantitative studies have demonstrated that diabetes stigma is associated with higher HbA1c levels in adults with T2D [6, 11, 22, 38, 41]. Only 2 of these studies obtained HbA1c values by laboratory measurement [38, 41]. One cross-sectional study using a validated survey found no significant association between stigma scores and self-reported HbA1c levels for those with T2D who were using insulin (N = 304) or were not using insulin (N = 328) when adjusted for age, gender, duration of diabetes, and number of diabetes-related complications [36]. One study using a validated survey demonstrated that higher stigma scores were associated with the presence of at least one complication [20]. There were no studies found assessing glucose time in range, DKA, or severe hypoglycemia in adults with T2D.

### Youth and Adolescents With Type 2 Diabetes

The SEARCH for Diabetes in Youth study, described earlier, examined diabetes stigma in youth and adolescents with T2D (N = 353), using an unvalidated survey [7]. This study demonstrated an association between diabetes stigma and higher HbA1c levels and the presence of retinopathy, when adjusting for race and ethnicity, sex, treatment plan, HbA1c, age, duration of diabetes, education level, and health insurance [7]. There was no significant association for DKA or severe hypoglycemia episodes in the prior year, or other long-term complications, such as dyslipidemia or nephropathy [7]. There were no studies found examining time in range and diabetes stigma in youth with T2D. There were also no studies found looking at parent-reported stigma and associations with clinical outcomes in youth with T2D.

### Summary

Diabetes stigma has been associated with elevated HbA1c levels in youth, adolescents, and adults with T1D and T2D [6, 7, 10-13, 17, 22, 36-41]. The causation of this association is unclear, given the cross-sectional nature of the studies. It is possible that people with diabetes who experience or perceive more stigma, conceal, delay, or forgo self-care behaviors (ie, blood glucose monitoring and/or medication administration) which in turn leads to higher HbA1c levels. The impact of stigma on self-care behaviors and psychosocial functioning is also a plausible mediator of the association of diabetes stigma, acute complications and chronic complications, independent of HbA1c levels. Future work is warranted regarding associations of diabetes stigma with acute complications in adults with T1D and T2D, impact of parent-reported stigma on youth diabetes outcomes, and clinical outcomes in youth and adolescents with T2D.

## Stigma, Psychosocial Outcomes, and Self-Care Behaviors

There is a larger body of research on diabetes stigma and psychosocial outcomes than for clinical outcomes. Diabetes stigma is associated with lower self-esteem [5, 18, 26, 27, 36] and decreased self-care behaviors [9, 11, 18, 33, 34, 36, 41] in adults with T1D and T2D, in addition to decreased medication adherence in adults with T2D [42], which may lead to elevated HbA1c levels and therefore increase the risk of complications. Diabetes stigma is also linked to depressive symptoms [5, 6, 11, 21, 26, 27, 36, 43], anxiety symptoms [26, 27, 36], diabetes distress (which is the emotional response to living with diabetes) [10, 11, 18, 26, 27, 34, 36, 38], and decreased general [1, 14, 43] and diabetes-related quality of life [1, 14, 38, 43] in people with T1D and T2D. Disordered eating behaviors is associated with diabetes stigma in adolescents and young adults with T1D and T2D [43], whereas lower resilience is associated with diabetes stigma in adults with T2D [20]. This decreased psychosocial functioning, which may be mediated by diabetes stigma, is likely to negatively impact adherence to treatment regimen, HbA1c levels, and risk of complications [25]. While the majority of studies are cross-sectional, one prospective study followed women with T1D and T2D (n = 193) for 6 months and showed that higher baseline self-stigma was indirectly associated with higher HbA1c levels via decreased quality of life [38].

### Summary

Diabetes stigma is associated with decreased self-care behaviors, which is likely to increase risk of complications and higher HbA1c levels. However, the directional relationship between stigma, self-care, and diabetes clinical outcomes has not been demonstrated. Lower psychosocial functioning has been associated with diabetes stigma and may be mediated through decreased quality of life. There is a lack of knowledge about the directionality and temporal relationship of psychosocial moderators of diabetes stigma.

## Socioeconomic Factors Associated With Stigma

Several socioeconomic factors have been associated with diabetes stigma. Lower household income has been associated with diabetes stigma in adolescents and young adults with T1D [7] and adults with T2D [8, 20]. A study from Iraq found a significant association between diabetes stigma and being unemployed in adults with T2D [8]. There have been mixed results on education level, with a large study in the United States (n = 5422) finding a higher prevalence of diabetes stigma for people with higher education levels [6] and 2 studies from China and Iraq demonstrating that diabetes stigma is associated with a lower education level [8, 20]. A study from Colombia on adults with T2D found that those who self-reported a lower socioeconomic status experienced more stigma [18]. The SEARCH for Diabetes in Youth study is the only study to report that food insecurity, another marker of socioeconomic status, is associated with higher diabetes stigma [43].

### Summary

Diabetes stigma is associated with lower socioeconomic status, although there are conflicting data on education level and stigma. This indicates populations with more socioeconomic stressors are at higher risk for experiencing diabetes stigma and efforts to address stigma in these populations should be prioritized.

## Cultural Factors Associated With Stigma

An individual's culture may impact how that person with diabetes experiences, perceives, or copes with diabetes stigma. Culture affects how people exhibit alternative thinking, feeling, and behaviors that may affect stigmatization and discrimination toward people with diabetes. Such differences may affect the definition of and manifestation of stigma [44]. A small body of research exists related to understanding stigma as a social construct in different cultures. For example, some cultures believe diabetes is contagious, some believe it is self-inflicted due to over-indulgence in unhealthy food, laziness, or lack of exercise, and other cultures perceive people with diabetes as being sick [45]. People with diabetes in many cultures are perceived as not suitable for marriage, especially young women [45]. If diabetes is common or seen as a normal lived experience in a community, then diabetes stigma may be less prevalent. At a more local level, having family members, friends, or colleagues with diabetes, either in their personal life, workplace, community support groups, advocacy groups, or summer camps, may also be protective against diabetes stigma.

### Summary

Cultural context and beliefs likely impact the prevalence of diabetes stigma in addition to how stigma is experienced. Little is known about how different cultures or communities drive or protect against diabetes stigma.

## Future Directions

While several validated stigma surveys exist, there are gaps in the current assessment tools. Future research should define a cutoff score for high frequency or clinically significant levels of stigma. Development of a validated survey for youth with diabetes less than 12 years old and youth less than 18 years old with T2D will be crucial for understanding and addressing diabetes stigma in these populations. Prevalence of diabetes stigma in youth and adolescents with T2D is currently unknown, making this population an important focus for future research.

The majority of studies are cross-sectional, which prohibits conclusion about directionality or temporality between HbA1c, acute and chronic complications, and diabetes stigma. Future research may include longitudinal work to assess the temporal and causal relationship between diabetes stigma, clinical outcomes, psychosocial outcomes, and self-care behaviors. An examination of moderators of diabetes stigma should include race, ethnicity, socioeconomic status, cultural factors.

Diabetes stigma is reported in the majority of people living with diabetes and is associated with worse clinical outcomes, making it an important aspect of diabetes care to address. An international consensus statement on addressing diabetes stigma details methods and areas for intervention, including within healthcare, policy, and advocacy [1]. This may include training on what it looks like to live with diabetes for teachers, healthcare workers, and employers. A larger scale approach with public service education should also be considered.

Supporting individuals with diabetes in recognizing, challenging, and coping with diabetes stigma may include building resilience [20], enhancing self-esteem, and nurturing social supports [1]. Social support may come from friends and family understanding what it means to live with diabetes, or from social contacts who live with diabetes and can empathize with each other. Cognitive behavior therapy has been shown to decrease diabetes distress, depression, health anxiety and increase quality of life and treatment adherence in people with diabetes [46]. Given that diabetes stigma is associated with decreased psychosocial functioning, cognitive behavior therapy is likely to have positive impacts on coping with diabetes stigma, although this has not yet been studied. Additionally, using person-first language in clinical practice and scientific journals can help change the narrative of living with diabetes [1, 47].

Several diabetes stigma assessment tools exist for different populations, although these have primarily been used in research. Incorporation of diabetes stigma surveys into clinical care would allow clinicians to assess and discuss the impacts of diabetes stigma when providing comprehensive diabetes care. By identifying individuals struggling with diabetes stigma, clinicians may empathize and validate their experiences, provide education on antidiscrimination laws and resources, and refer to cognitive behavior therapy, resiliency building programs, diabetes camps, and/or peer support groups for people with diabetes.

## Conclusion

While diabetes stigma has been a longstanding lived experience for people with diabetes, research in the field is limited but expanding within the last several years. The prevalence of diabetes stigma has been reported to be more than 70% in adults with T1D and T2D [3, 4, 6, 8-11], 98% in youth and adolescents with T1D [12-14], and is unknown in youth and adolescents with T2D. Diabetes stigma has been associated with elevated HbA1c levels [6, 7, 10-13, 17, 22, 36-41] and psychosocial complications [1, 5, 6, 9-11, 14, 18, 20, 21, 26, 27, 33, 34, 36, 38, 41, 43] in adults, adolescents, and youth with T1D and T2D. A smaller number of studies have demonstrated that diabetes stigma is associated with the presence of chronic complications in people of all ages with T1D and T2D [7, 10, 20] in addition to acute complications of DKA and severe hypoglycemia in adolescents and young adults with T1D [7, 12, 40]. Addressing diabetes stigma will require several strategies in public policy, advocacy, and public education, and training in healthcare, workplaces, schools, and childcare centers, in addition to the diabetes clinic.

## Data Availability

Data sharing is not applicable to this article as no datasets were generated or analyzed during the current study. The findings and conclusions in this report are those of the authors and do not necessarily represent the official position of the Centers for Disease Control.

## Abbreviations

DKA: diabetic ketoacidosis
DSAS-1: Type 1 Diabetes Stigma Assessment Scale
DSAS-2: Type 2 Diabetes Stigma Assessment Scale
HbA1c: glycated hemoglobin
T1D: type 1 diabetes; T2D type 2 diabetes
